# PROJECT ECHO FOR NURSING HOMES: A PATIENT-CENTERED, RCT TO IMPLEMENT INFECTION CONTROL BEST PRACTICES IN NHS

**DOI:** 10.1093/geroni/igac059.920

**Published:** 2022-12-20

**Authors:** Emily Heilbrunn, William Calo, Erica Francis, Lan Kong, Ellie Hogentogler, Abbey Fisher, Nancy Hood, Jennifer Kraschnewski

**Affiliations:** Project ECHO, Penn State University College of Medicine, Hershey, Pennsylvania, United States; Penn State College of Medicine, Department of Public Health Sciences, Hershey, Pennsylvania, United States; Penn State College of Medicine, Department of Medicine, Project ECHO, Hershey, Pennsylvania, United States; Penn State College of Medicine, Department of Public Health Sciences, Hershey, Pennsylvania, United States; Penn State College of Medicine, Department of Medicine, Project ECHO, Hershey, Pennsylvania, United States; Penn State College of Medicine, Department of Medicine, Project ECHO, Hershey, Pennsylvania, United States; University of New Mexico, Project ECHO, Albuquerque, New Mexico, United States; Penn State College of Medicine, Department of Medicine, Hershey, Pennsylvania, United States

## Abstract

Nursing homes (NHs) have been devastated by COVID-19. Only 3% of designated infection preventionists in NHs have taken a basic infection control course. Little is known about the implementation of effective infection control practices in NHs. This study utilizes Project ECHO (Extension for Community Health Outcomes), an evidence-based tele-mentoring model, to connect subject matter experts with NH staff to proactively support evidence-based infection control guideline implementation. This study will determine how guidelines can be implemented effectively in NHs, including reducing COVID-19 diagnoses and improving other key patient-centered outcomes (e.g., quality of life) NHs (N=136) were recruited and assigned to ECHO or ECHO Plus using a randomized design. A multi-pronged approach to improving infection control and emergency preparedness in NHs is important. The ECHO model has significant strengths allowing for remote learning delivered by a multi-disciplinary team and utilizes case discussions that match the context and capacity of NHs.

